# A case report of massive retrosternal goiter in a 54‐year‐old woman with symptoms of head and neck swelling and dyspnea

**DOI:** 10.1002/ccr3.8918

**Published:** 2024-05-22

**Authors:** Shahabaddin Sorouri, Samira Akbarianrad, Maryam Naseri

**Affiliations:** ^1^ Lung Diseases Research Center Mashhad University of Medical Sciences Mashhad Iran; ^2^ Internal specialist Mashhad University of Medical Sciences Mashhad Iran; ^3^ Department of Pediatrics Mashhad University of Medical Sciences Mashhad Iran

**Keywords:** cervical thyroidectomy, mediastinal mass, retrosternal goiter, sternotomy

## Abstract

Anterior mediastinal mass often is serious and its diagnosis requires a comprehensive evaluation involving imaging studies, pathological analysis and consultation with a multidisciplinary team involving radiologist, thoracic surgeons, and oncologist.

## 1 INTRODUCTION

Retrosternal goiter (RSG) is defined as thyroid enlargement with the largest mass along dermal sternum from the neck to the substernal part progressing below the thoracic inlet and is biologically inseparable from nodular goiter.^1^ Although there are different definitions for RSG, the currently accepted definition is the presence of more than 50% of the thyroid gland mass below the thoracic inlet.^2^ Depending on the criteria used to define RSG, its incidence varies from 0.2% to 45% of thyroidectomy patients.^3^ Although goiters are diffuse at first, but over time, factors cause them to become nodular. Iodine deficit is generally accepted as a major factor that contributes to the increase in thyroid nodulation. Various environmental and genetic factors, each with their own effects, play a role in the abnormalities of the thyroid gland, but contribution of various factors ultimately trigger nodular formation, especially if a person is genetically predisposed.^4^ In huge goiters, there is a greater possibility of mediastinal extension, and this is more likely to happen in the fifth and sixth decades of a person's life. RSG is more likely to be on the left side, and rarely left cervical goiter descends to the right side of the chest.^5^ This manuscript reports a rare case of 54‐year‐old female who had a huge RSG which has rarely been observed. The patient was finally treated with sternotomy and cervical thyroidectomy.

## 2 CASE PRESENTATION (CASE HISTORY/EXAMINATIONS)

A 54 years old woman with past medical history of HTN was presented to our hospital with chief complaint of dyspnea and facial swelling and redness when standing up. The patient's symptoms had started gradually 3 months before she went to our hospital and had become more severe over time.

## 3 METHODS (DIFFERENTIAL DIAGNOSIS, INVESTIGATIONS AND TREATMENT)

**FIGURE 1:**
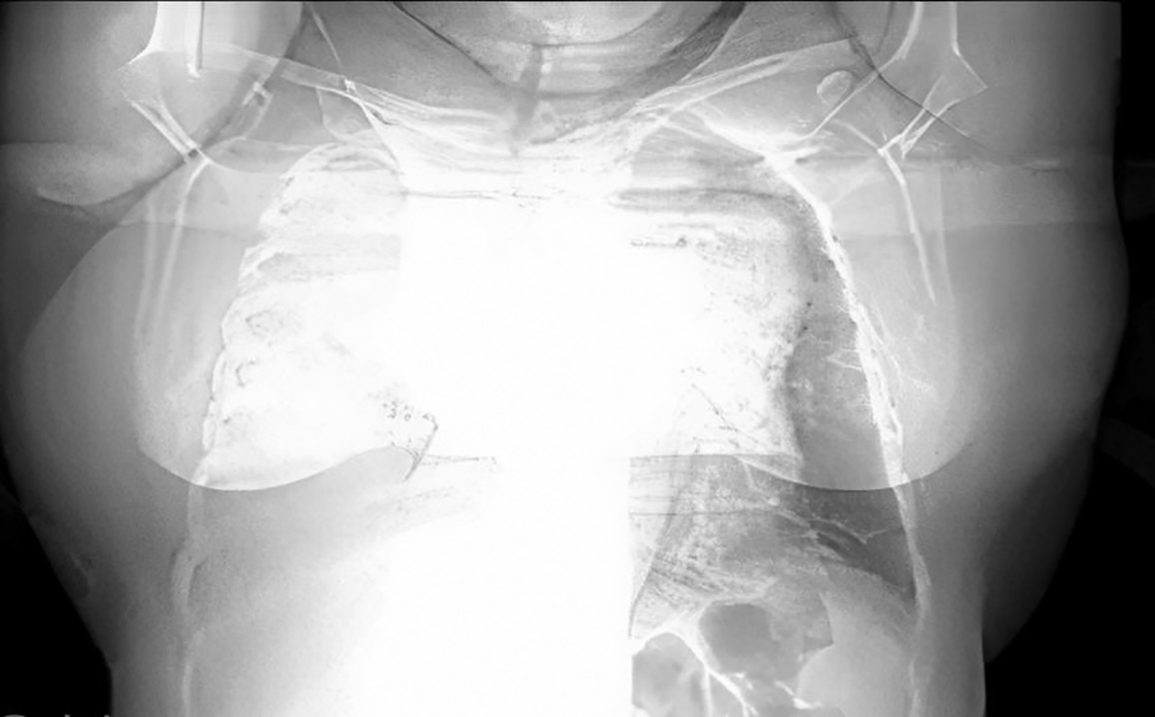
Chest X‐ray, deviated trachea.

**FIGURE 2:**
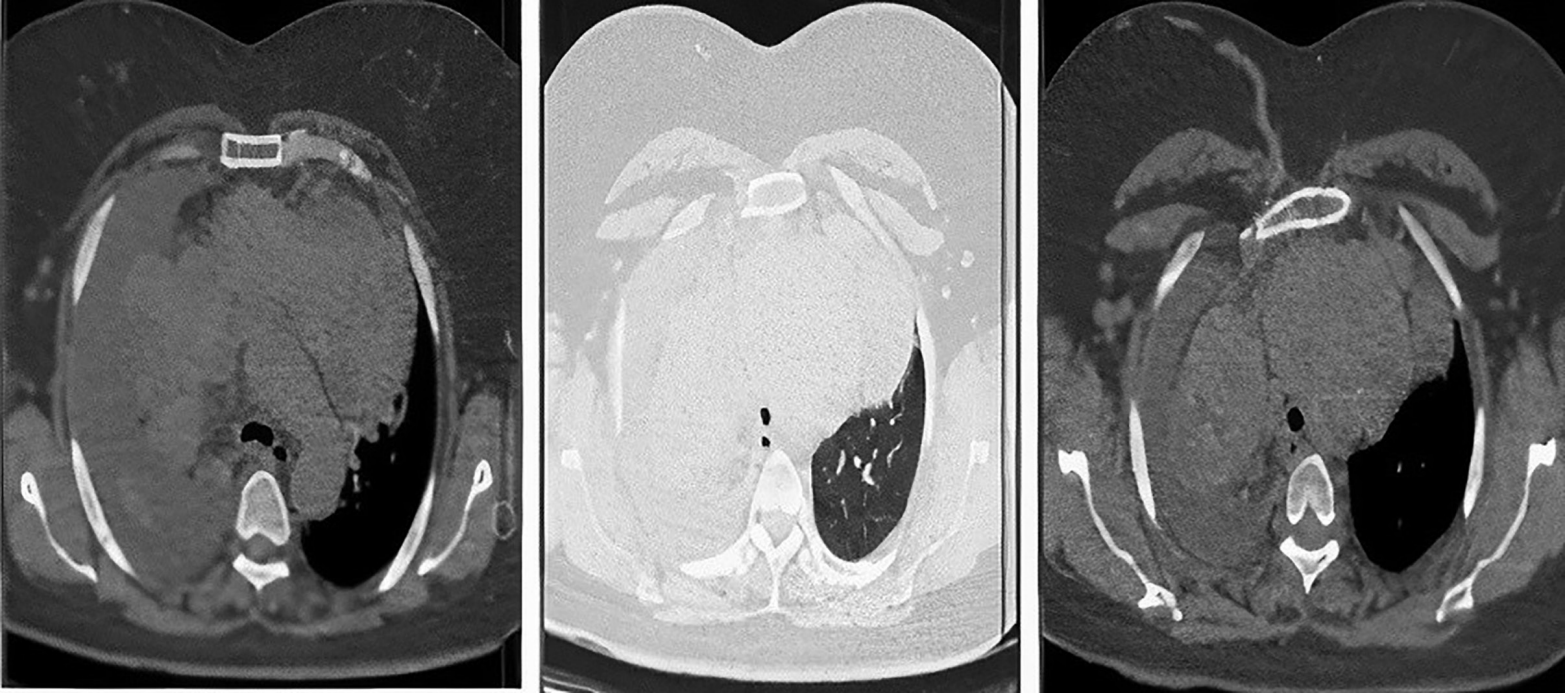
Lung high‐resolution computed tomography (HRCT), pleural fluid.

Chest X‐ray (CXR) and lung high‐resolution computed tomography (HRCT) were performed in the first stage (Figures 1 and 2). According to the pleural fluid reported in the computed tomography (CT) scan, a diagnostic tap of the pleural fluid was performed, which was reported to be exudative with lymphocyte predominance. Then a biopsy of the pleura was performed and neoplasia was not identified.

**FIGURE 3:**
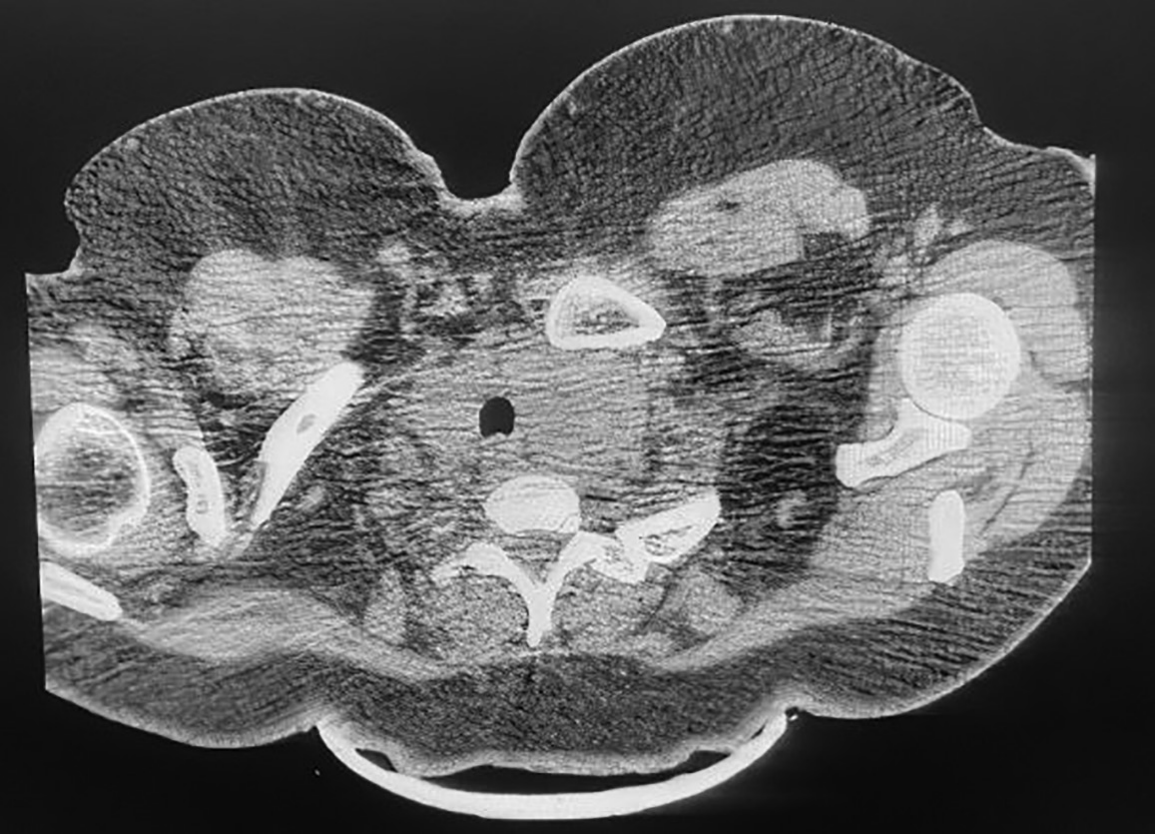
Computed tomography (CT) scan of the neck showing a large retrosternal goiter (RSG).

In the following, according to the ultrasound performed on the soft tissue of the neck, the right thyroid lobe measured 82 × 62 mm and the left thyroid lobe measured 74 × 42 mm, which extended to the upper part of the mediastinum. As a result, the trachea had been displaced posteriorly which can primarily indicate multinodular goiter. Figure 3 shows CT scan of the neck. The parenchymal echo of both lobes was heterogeneous. Color Doppler ultrasound of the neck was performed and considering the patient's lack of positioning and dyspnea, it could be checked only to some extent. Accordingly, the image of the heterogeneous hypoechoic focus corresponding to the left thyroid had a compressive effect from the medial part and the left carotid artery. Investigation of the origin of the left carotid artery was not possible due to the mentioned mass. A normal arterial flow with a normal spectral wave and PSV in the normal range was seen in the common, internal, and external carotid arteries. No image was seen in favor of plaque or parietal calcification in the wall of the common, internal, and external carotid arteries. It was not possible to examine the vertebral arteries due to the patient's lack of positioning and the patient's dyspnea. IMT of the left side was 0.8 mm and IMT of the right side was 0.7 mm. Considering that the thyroid origin of the above‐mentioned mass was possible, a radioiodine scan was first requested and the result was huge RSG. To confirm the diagnosis, sampling was done using through‐the‐needle biopsy (TTNB), and due to the closeness of the lesion to the nearby vessels, a CT scan with contrast was performed first, and then a sample was taken from the lesion under CT guidance.

**FIGURE 4:**
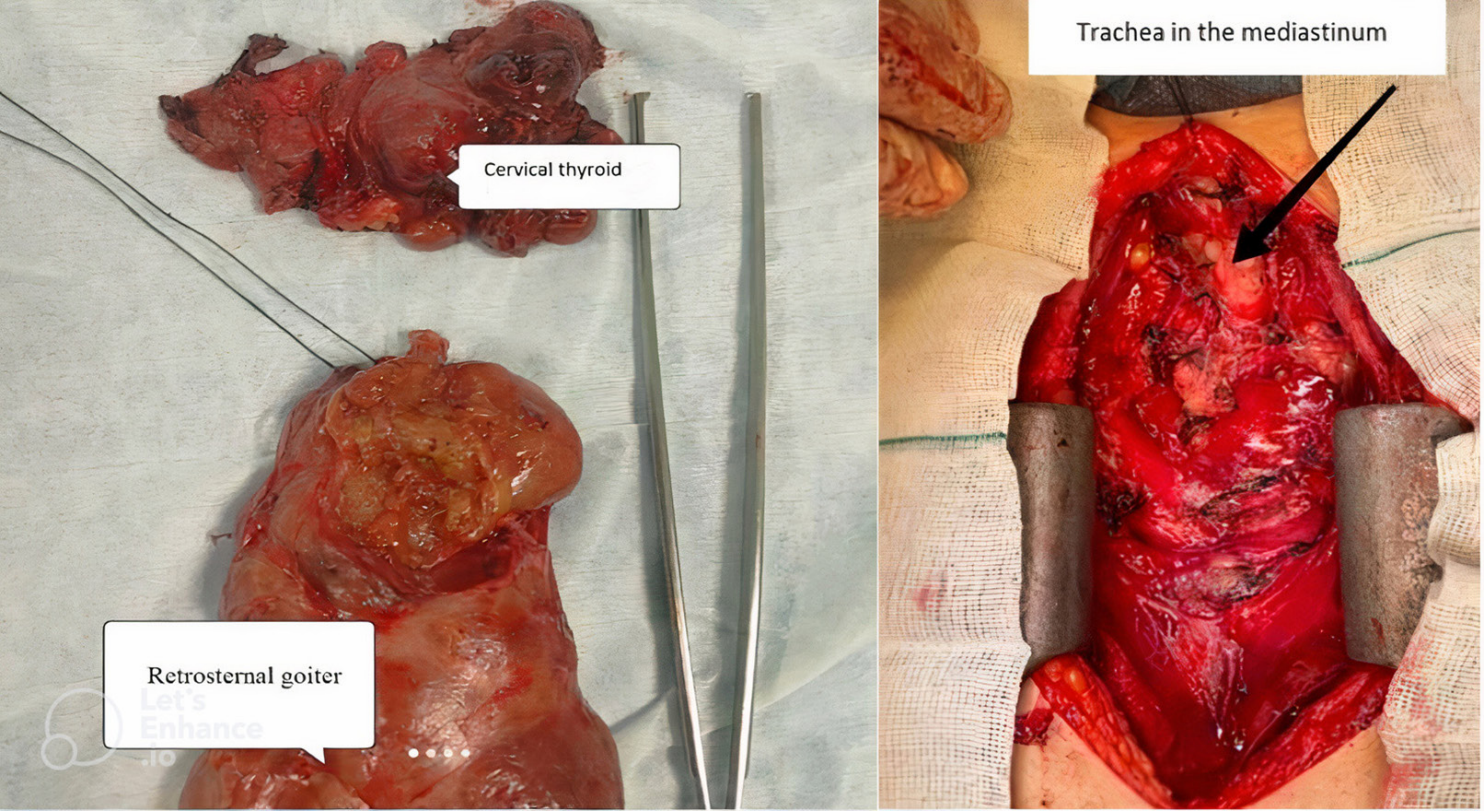
Surgical images, left side image: removing the thyroid gland along with the retrosternal mass, right side image: Compressed trachea.

In spiral lung CTs with contrast a large multinodular with heterogeneous mass with enhancement in the post‐contrast phase and with central necrosis in the upper and anterior mediastinum and with classification with a compressive effect on the left brachiocephalic vein and branches from the aorta which seems to originate from the left thyroid lobe, was evident. Which can be the cause of massive RSG. Massive pleural effusion was evident in the right hemithorax with near total collapse of the lower lung. Mild pericardial effusion with a maximum thickness of 6 mm was seen. Heterogeneity was seen in the parenchyma of the right lobe and hypodense nodule in the right lobe of the thyroid. The pathological answer was multinodular goiter. Then, the patient underwent thyroidectomy surgery (Figure 4, operative pictures), where a complete median sternotomy was performed.

## 4 RESULTS (OUTCOME AND FOLLOW‐UP)

Superior vena cava (SVC) was separated from the mass and preserved. The pleura was opened on both sides and the effusion was suctioned on both sides. Next, the phrenic nerve was explored and preserved on both sides. Cervical thyroidectomy was performed with the preservation of the recurrent nerve on both sides and parathyroids. The patient recovered after surgery and was discharged.

## 5 DISCUSSION

The natural course of goiter is characterized by a slow progression of symptoms and progressive enlargement with obvious swelling of the neck, pressure marks, or secondary hormonal disturbances.^6^ Dyspnea is the most common symptom in RSG, and orthopnea, dysphagia, and dysphonia are other common symptoms.^7^ Occurrence of compressive effects on neurovascular structures and development of SVC syndrome are rare.^8^ Neck swelling, dyspnea and SVC syndrome were observed in our case.

Due to the existence of different definitions of RSG, preoperative investigations can help to identify patients who need surgical resection. CT can show the effect of RSG on the mediastinal components and the occurrence of extension, as well as the pressure on the trachea or esophagus.^8^ In the present study, multinodular goiter was identified using CT. The incidence rate of RSG in women is higher compared to men and the ratio is 3:1.^9^ Similar to the present study, which was about a female case, there were two similar case reports of huge RSG in women. In one case the patient was a 32‐year young woman who had history of thyroidectomy due to goiter and a 1‐year large mass on front of the neck. Diagnostic studies showed a massive RSG, which caused the deviation of the trachea. In this case, the goiter was surgically removed through a single neck incision.^2^ Another study reported nodular goiter with huge retrosternal extension in a 55‐year‐old female. Like present case, removing the mass through conventional approach was impossible and hence total thyroidectomy was performed using a combined cervical/sternotomy approach.^5^ In case of present research, cervical thyroidectomy and median sternotomy were conducted. In the study of McKenzie et al., RSG was observed in the posterior mediastinum and thyroid density on CT imaging were introduced as independent risk factors for an extra‐cervical approach and need for sternotomy.^10^

Regarding thyroidectomy approaches, it is recommended to perform total thyroidectomy rather than subtotal thyroidectomy to prevent the recurrence of RSGs. Cervical approach is suitable for goiter removal in more than 95% of cases.^11^ In conditions such as the presence of very large RSGs, invasive cancer, and previous cervical thyroidectomy, sternotomy or thoracotomy may be recommended in patients. In symptomatic RSG surgery, it should be noted that radioiodine ablation leads to airway obstruction in the acute possibility of radiation thyroiditis. Also, a large percentage of RSGs tend to regrow, and about a quarter of RSGs contain malignancy. The transcervical approach is considered a safe approach in removing about 90% of RSGs.^4^

## 6 CONCLUSION

Although most cases of RSGs can be treated with a transcervical approach, in cases such as a very large size of goiter and its growth towards the mediastinum that was also observed in our case, sternotomy must be adopted.

## AUTHOR CONTRIBUTIONS

**Shahabaddin Sorouri:** Data curation; formal analysis; supervision. **Samira Akbarianrad:** Conceptualization; investigation; writing – review and editing. **Maryam Naseri:** Investigation; writing – original draft.

## FUNDING INFORMATION

We received no funding for this case report.

## CONFLICT OF INTEREST STATEMENT

The authors declare that they have no conflicts of interest.

## CONSENT

Written informed consent was obtained from the patient to publish this report in accordance with the journal's patient consent policy.

## Data Availability

Data sharing not applicable to this article as no datasets were generated or analyzed during the current study.
